# Science, Sexual Difference and the Making of Modern Marriage in American Sex Advice, 1920–40

**DOI:** 10.1111/1468-0424.12591

**Published:** 2022-01-13

**Authors:** Sarah L. Jones

**Affiliations:** ^1^ Department of History University of Bristol Bristol, United Kingdom of Great Britain and Northern Ireland

## Abstract

This article considers discussions of sexual difference in a range of popular prescriptive texts published between 1920 and 1940 in order to explore the relationship between science and American marital advice literature. It demonstrates the particular role science played in shaping, legitimising and enforcing changing discussions about what ‘good sex’ should look like in contemporary advice – supporting a hierarchy of sexual activities and desires that privileged a particular version of marital heterosexual expression. Through this, it also interrogates the ‘popular’ version of sexual science being consumed by the American public at this time. In addition to adding new perspectives to our understanding of contemporary advice and its relationship with science and medicine, it will also act as a provocation for further research into the ways the public engaged with sexual science in early twentieth‐century America.

In the early twentieth century, advice about what a good American marriage should look like was changing. As numerous scholars have shown, earlier assertions about the necessity of continence, self‐control and procreative marital sexuality gave way in this period to calls for a new, sensual heterosexuality explicitly based on erotic fulfilment apart from reproduction.^1^ Advice writers began to emphasise that a mutually satisfying erotic life was imperative to achieving marital bliss and, rather than warning of the dangers of sexual over‐indulgence, now actively encouraged couples to spend time and energy on their sexual lives separately from reproductive intent. Reflecting a changing cultural climate in which impetus was increasingly placed on individual fulfilment, many advice authors now stated that mutually satisfying, emotionally invested and consistently orgasmic sex was healthy, natural and necessary.^2^

Contemporary advice authors emphasised that a shift towards what was meant to be a fairer and more pleasurable marital experience represented progressive change, ensuring the creation of happy and harmonious marriages. Authors were particularly keen to emphasise how these changes signified a move away from the repressive, patriarchal norms of Victorian marriage, guaranteeing sexual equality and a shift away from a ‘world ruled exclusively by men, into a domain mutually controlled’.^3^ But such congratulatory proclamations about the benefits of eroticised heterosexuality were taking place within the context of key socio‐economic changes, including concerns about the changing social and political role of women, a falling birth rate among the white middle classes, increasing fears of homosexuality and broader anxieties about the potential collapse of orthodox marriage.^4^ In this context, advice writers’ visions of ‘ideal’ sexuality have been read as serving to reinforce boundaries around what constituted appropriate sexual expression: while advice now recognised that sexual culture was changing, it nevertheless served to privilege marriage as the only legitimate site of sexual activity, support ideas about male superiority, reinforce racial hierarchies and notions of white supremacy and condemn anyone – the unmarried, the ‘frigid’ or the homosexual – whose sexual activities and proclivities fell outside of the boundaries of marital heterosexuality.^5^

To an extent, science is already part of the broad story historians have told about what Jessamyn Neuhaus calls the changing ‘sexual script’ of early twentieth‐century America.^6^ Scholarship has, for example, explored attempts to redefine the boundaries of desirable marriage as a tool of positive eugenics, investigated the influence of Freudian psychoanalysis on contemporary sexual mores and also looked at the growing authority of the professional sexologist in American society and culture.^7^ But while we know that science, broadly understood, was a key part of conversations about sex at this time, I would argue that historians’ specific treatment of what we might think of as ‘popular sexual science’ has often been quite superficial. Histories of advice, for example, have often glossed over the specific scientific content of marriage guides in the first half of the century, commonly treating science as a common‐sense part of contemporary sexual discourse that requires little in‐depth interrogation or contextualisation.^8^ In many of these narratives, what it means for prescriptive texts to treat sex ‘scientifically’ is explored only superficially; a peripheral concern in research often more focused on the gender politics of contemporary advice.

Historians of sexology, on the other hand, have been even more dismissive of the relationship between science and sex advice. Many argue that the first few decades of the century were something of a lacuna for popular sexual scientific discussions in which people were, at best, only able to access very basic scientific information about topics like venereal disease and sexual selection.^9^ While increasing scholarly attention is being paid to the richness of early American sexological research, historians like Janice Irvine and Miriam Reumann have treated mid‐century as a kind of watershed moment in which sexual science initially captured the public imagination, encouraging them to think ‘scientifically’ about their sexuality for the first time.^10^ Those who do chart the relationship between science and advice often tend to assume that scientific knowledge about sex was only produced by key figures in contemporary American sex research and only circulated within elite medical spaces or published with a specialist medical or legal audience in mind.^11^ As Laura Doan has argued, in such accounts, advice is usually only seen as the end point of a ‘top‐down’ model, in which knowledge ‘trickles down’ from the work of a set of monolithic sexological figures.^12^ But, as recent research around popular science has effectively shown, this model of elite, ‘top down’ science is problematic – ignoring as it does the complex ways scientific knowledge was produced and consumed at this time, and falling back on assumptions of ‘a kind of downward or outward flow of knowledge from scientist to public’.^13^

This article therefore aims to add new insights to our current understanding of ‘popular’ sexual science and early twentieth‐century sex advice. It will begin to explore in detail the version of science being sold to, and consumed by, the American public through popular print. In short, it will investigate what it looked like to write ‘scientifically’ about sex for the public at this time. To do so, the article will look at a range of different types of advice that would have been available to many readers across America in this period. Such texts were sold in bookshops and newsstands, but were also available with relative ease through mail order, ranging in price from a few cents for a pamphlet or magazine, to a couple of dollars for a more substantial book.^14^ In addition to some well‐known sex advice books published in the opening decades of the century, it will also explore other important widely available texts often overlooked in accounts of both sexual science and sex advice. Kansas‐based publisher Emanuel Haldeman‐Julius's hugely successful series of ‘Little Blue Books’, for example, included a range of broadly scientific information and advice regarding sex. From their launch in the early 1920s, sex and marriage advice books dominated Haldeman‐Julius’ sales figures, and he boasted of selling tens if not hundreds of thousands of his most popular sex titles.^15^ These books were sold through mail order at five cents each and reached wide audiences across the social spectrum – Jay Gertzman states that they were as likely to have been read by well‐to‐do readers of the New York Times as they were by the middle‐ and working‐class readers of low‐priced general interest magazines.^16^ It will also look at early editions of *Sexology* magazine, founded by science fiction editor Hugo Gernsback in 1933. Aimed at readers ‘eager for real facts and for knowledge’, it included an eclectic array of articles on all aspects of human sexuality written by a diverse range of authors.^17^ Sold for a modest twenty‐five cents and available through mail order, it was successful enough to stay consistently in print all the way through to 1983.

The sources are replete with discussions, allusions and promises of ‘scientific’ information about sex. Authors boasted at length about their apparent scientific pedigree, claiming that science could shed light on every aspect of American's sexual lives. One advice writer hailed this new literature as a ‘wave of revolt … sweeping over the Western world’, doing away with outdated prudery and ushering in a ‘flood of new light’ around all sexual subjects.^18^ However, in this article, I am going to use a particular case study for my investigations – assertions of the importance of sex difference, and specifically advice, which emphasised that to achieve a successful sexual life men and women should play different but complementary sexual roles. As existing research has shown, these discussions of gender‐appropriate sexual behaviour were a key aspect of contemporary advice, making prescriptive literature an important site of ‘gender contestation’ for the white middle classes at this time.^19^

In doing so, it will outline some of the specific forms and functions of science in this popular literature, consumed by millions of readers looking for answers about sex at a key point in American sexual history when new ideas about ideal marriage were emerging. As well as exploring how and why ‘science’ was marshalled to support notions of sexual difference, it will suggest that the scientific content of this kind of advice was usually more rhetorical than technical. Commonly embracing what Max Saunders calls a ‘scientific spirit’, authors blended materials and approaches from a broad range of disciplines without spending much time working with specific theories or methodologies.^20^ While Wendy Kline is therefore right, to an extent, to claim that notions of sexual difference in contemporary advice were simply ‘dressed in the garb of science’, this article will further deepen and complicate these conversations about what it meant to do sexology in public before the works of researchers like Kinsey and Masters and Johnson bled into mainstream American culture.^21^ Through explorations of sexual difference in mass‐produced sex advice, it will demonstrate that the version of sexual science consumed by the reading public in early twentieth‐century America was a curious mash of content, old and new approaches, and diverse – and sometimes unexpected – points of authority.

The idea that men and women had different sexual characters and therefore distinct roles to play in the marital bedroom was a key focus of contemporary marital advice. Advice texts promised husbands and wives valuable insights into the unique, sometimes seemingly alien sexual nature of their partners; knowledge that authors promised would help ensure happy and harmonious marriages. One contemporary advice author, for example, asserted that ‘clear knowledge is needed: knowledge of the differences between the sexual constitutions of men and of women and the adjustment problems which arise out of these differences’, as this was key to achieving a mutually pleasurable sexual life, and consequently a successful marriage.^22^ It was not, of course, new or particularly radical to argue that the sexes were different. As numerous scholars have shown, there is a long history of theories of sexual difference that have been used in diverse ways to support or challenge notions of ‘separate spheres’ for men and women.^23^ In early twentieth‐century sex manuals like those studied here, though, ideas of sexual difference were being tied much more closely to eroticism and actual sexual behaviour, used less to talk about men and women and their role in society generally, and more about how the different sexual natures of husbands and wives should play out in the marital bedroom. In these texts, advice authors claimed that a successful sexual life, and therefore a happy marriage, specifically hinged on a couple's ability to recognise the different but complementary sexual natures of each partner and to live up to the gendered expectations about appropriate sexual behaviour.

While the messages about male and female sexuality were diverse, authors generally agreed on the ‘activity’ of male sexuality versus the sexual ‘passivity’ of women. Men were assumed to have a potent and dynamic sexuality in need of control and refinement; women, on the other hand, were acknowledged as powerfully sexual beings, but at the same time authors emphasised that this sexuality had to be coaxed to the surface, stirred into existence by a patient and skilled husband.^24^ A wife was expected to be sexual but not *too* sexual; coyly welcoming her husband's sexual advances but lacking any real sexual impetus of her own. For example, reflecting widespread assumptions, Dr Wilfred Lay's *Confidential Chats with Husbands* spoke of ‘true masculinity’ as ‘the innate impulse to dominate, to be the creative factor, to be productive’ in the bedroom; a tendency he contrasted with ‘true femininity’, that is, ‘the innate impulse to be dominated, to be led, to be influenced, to be given emotions by direct action’.^25^ In these texts, women who did experience sexual arousal too independently of their husband's efforts were presumed to be experiencing ‘pathological’ or ‘abnormal’ eroticism, signifying, too, a tendency towards criminal activity or promiscuity.^26^ Advice writers mostly claimed that recognising these apparent differences and living up to these expectations would ensure more harmonious sexual experiences, and indeed many women may have felt their marital lives were improved by taking on such prescriptions. However, these ideas can also be seen to support a rather restrictive view of what sexuality should be that reflects prominent contemporary anxieties about gender and sexuality. Sex ‘done right’ was to be heterosexual, marital and, for women, only appropriate when encouraged by her husband.

Readers of these texts were told that such differences could be best explained using science and were promised that for just a dollar or two they could learn ‘all the secrets of sex’, through an exploration of ‘scientific pathological facts’.^27^ The science on display here, though, can probably best be characterised as rather woolly. Indeed, authors often turned to quite generalised and non‐specific discussions of physiology, anatomy and biology to articulate and rationalise notions of activity and passivity, binding assertions about different sexual characters to assumptions about the specific workings of sexed bodies. Despite the fact, readers were now told sex should be thought of separately to reproduction, it was especially common for advice texts to ground such claims in somewhat vague theories about what their authors argued were the biological realities of reproductive bodies. Amidst fundamental social, political and cultural shifts around sex and gender that were occurring at this time, popular advice authors continued to marshal the cultural power of science to make their case – to argue, for example, that biology dictated that the workings of sexed bodies, and therefore the ‘natural’ relations between the sexes were to a significant extent fixed and unavoidable.

For women, this meant there were frequent, broad‐brush discussions of the importance of the ‘characteristic development and functioning of her generative system’, or her role as the creator and nurturer of life.^28^ Despite the lengths authors went to argue that marital sex should be seen as being about more that the production of children (and indeed most manuals included favourable discussions of birth control and family limitation by the 1920s), understandings of woman's sexuality were still often tightly linked to her specific reproductive function.^29^ The most common iteration of this was in the assumption that women were naturally more sexually conservative and slow to react to sexual stimuli due to what one author called the ‘responsibilities of motherhood, whether actual or potential’.^30^ Authors argued that a wife's reproductive role was much more complex and important than that of her husband, leading to a sexual nature that was more emotional and reactionary. As one author stated, for example, the woman was the ‘regulator of the process … the balance wheel of the whole machinery’ of human life – a ‘burden’ and responsibility that he claimed mandated sexual passivity.^31^ It was asserted that the physiological strains of pregnancy and childrearing dictated a tendency for women to be ‘intuitively reserved and hesitant in the sexual relations’, with women's sexual character shaped by a ‘natural defensive armor of coyness, indirectness and a tendency to delay the consummation of the sexual act’.^32^ It was not that women were completely lacking in sexual desire. In fact, authors went to great lengths to show that ‘women's sexual needs and capacities are wholly comparable to that of men’, with one text describing the female sexual instinct as comparable to ‘constantly smouldering volcanoes’.^33^ ‘Pure sexual feelings’, one manual on sexual physiology claimed, was a ‘fundamental law’ of woman's nature, without which ‘she would not be a womanly woman’.^34^ However, authors turned to the language of biology and physiology to argue that they had a powerful sexual nature of a different kind – ‘not so conspicuously aggressive, and passive, therefore, in a relative sense’ to that of their husband.^35^ It was therefore often argued that, in most women, sexual desire and expression could only be spurred on by careful attention from a husband she considered a companion and protector.^36^ This particular sexual character was ‘not a question of choice or will’, but instead mandated by ‘woman's physiological being, with its highly organized nervous system’.^37^ Regardless of new options for birth control or broader changes to the status of women in society, advice authors contended that the innate biological possibility of motherhood for women commanded her ‘protective reticence’, shaping her particular, passive, sexual nature.

Men, on the other hand, were said to have evolved to be women's biologically defined opposites. While a wife was assumed to be ‘slow … and passive’, her husband was characterised as innately ‘swift, ready and active’, both in and out of the bedroom.^38^ While authors continued to advocate for relative sexual moderation, they nevertheless argued that, from around the onset of puberty, sexual desire assumed ‘control of [man's] entire nature, becoming paramount to all else’.^39^ Mirroring the discussions around female sexuality outlined above, justifications for claims about the ‘active’ sexual nature of men were often grounded in ideas about their particular role in human reproduction; specifically, they were often linked to man's responsibility to fertilise – to spend and impregnate, rather than retain and nourish.^40^ While women's reproductive role and more complex physiology were seen to dictate a lack of sexual aggression, it was argued that men were characterised by simpler, less complex types of desire; a blunter sort of instrument, more directed towards prolificacy and frequent sexual expression.^41^ Regardless of a man's reproductive intent, his innate urge to fertilise was said to ensure a sexual nature that was dynamic and vigorous: as Clement Wood stated, a man's ‘biologic imperative commands him, in season and out of season, always and under all circumstance, to do his fertilizing duty, and never on any pretext to allow an opportunity to escape to infuse into the old hereditary trunk of his species, the female, the new life that is in him’.^42^ As with discussions of female sexual nature, generalised ideas about man's particular biological make‐up and his specific role as the fertiliser of seed also shaped assumptions about his innate and unavoidable sexual character. While women, burdened by the possibility of motherhood, were to be the passive partner, advice authors asserted that man was to be burden free, always ready and willing, and actively looking for opportunities for sexual activity – naturally inclined to his appropriate role as the sexual aggressor in the marital bedroom.

Often, advice authors’ discussions of the different but complementary sexual natures of men and women used these kinds of light‐touch, simplified or ambiguous references to biology, physiology and anatomy. Pitched at a non‐expert audience, such broad claims about the workings of the body would have been accessible to most readers, lending claims about the immutably ‘active’ and ‘passive’ sexual natures of men and women a general air of rationality, respectability and scientific authority. By asserting their information was ‘factual’ and ‘scientific’, authors made their claims seem simultaneously modern and progressive, and timeless and innate. Evolutionary biology was a particularly frequent reference point in many advice texts, and it was common for texts promising advice on modern marriage to start with descriptions of how humans had evolved over millennia to be sexually differentiated. While they acknowledged modern influences, they were often focused on demonstrating how their visions of proper masculine and feminine sexual roles were the outcome of evolutionary processes. Wood's *The Evolution of Sex*, for example, charted a long history from asexual reproduction at the origin of life, the developing sexual behaviours of plants and animals, through chaotic periods of female and male rule, to the apparent emergence of modern, romantic marital norms.^43^ Touted as the endpoint of a long process of differentiation and specialisation, authors like Wood argued that new claims about the importance of mutual pleasure, sex apart from reproduction and the complementarity of the sexes were markers of highly evolved humanity.^44^ While Kimberly A. Hamlin has shown that these kinds of evolutionary theories could be interpreted in different ways and contained ‘the seeds of radical interpretations’, the version of human evolution played out in advice texts usually had the same heterosexual, romantic, consistently orgasmic married couple at its apex.^45^

However, reflecting the prominent and fractious debates around the biological roots of sexual difference occurring in scientific circles in the late nineteenth and early twentieth centuries, authors did not use a single set of theories to make their claims.^46^ Indeed, authors employed a diverse mix of both old and new ideas in their work, often adapting these to fit the points being made. Some authors, for example, argued that the roots of sexual difference lay in older metabolic theories that had been used in various ways to debate sexual difference and the position of married women in the Victorian era.^47^ Drawing from the work of scholars like Patrick Geddes and J. Arthur Thompson (whose 1889 book *The Evolution of Sex* was one of the most influential texts on sexual difference in the late nineteenth century), some twentieth‐century advice texts now claimed that innate differences between the sexes, and particularly the gendered expectations about their behaviours in the marital bedroom, could be justified through explanations of the unique ways male and female bodies used energy. Relying on these older theories, authors claimed that women's sexual passivity was linked to her supposedly ‘anabolic’ nature; tied up, again, with her potential for maternity as authors argued a woman was ‘more given to repose and, for good biological reasons, the conservation of energy’.^48^ Men, on the other hand, were their natural opposites; as the ‘catabolic’ element of the race, they were seen to be characterised by an active, vigorous sexuality borne of their ‘instinctive urge for action, and the utilization of energy’.^49^

However, mirroring the growing social and cultural influence of endocrinology in the first half of the twentieth century, many advice writers grounded their arguments about the inherent dissimilarities between the sexes in discussions of ‘internal secretions’, emphasising that sexual difference was chemically commanded by a complex interplay of hormones.^50^ In *Sexology* magazine, for example, William Lemkin assured readers that ‘extensive investigation’ by scientists had proved that the ‘primary dictators of the process by which male and female are differentiated, if not created, are the interstitial glands’.^51^ Though he did not outline any specific research, he nevertheless confidently argued it was in these secretions that the ‘definite chemical basis of masculinity and femininity’ could be found; the hormones working, in his view, as a device that ensured ‘specialization’ and ‘differentiation’ between the two sexes.^52^ These hormonal differences often supported notions of male activity and female passivity in much the same way at the metabolic theories of energy. William J. Robinson argued the internal secretions of testicles would act as an ‘energizer of the whole body, stimulating the brain, the nervous system, the muscles, etc.’, while also actively producing substances like ‘*libidogen*, whose action is to *stimulate the sexual desire*’.^53^ On the other hand, the hormones of women were also said to produce a sex tension, though this was assumed to be ‘more diffused, less localized in the sex organs than it is in men, and hence does not so frequently result in specific desire and excitement’.^54^ Concepts of sexual difference and complementarity, seen as a key aspect of new guidelines for achieving a happy marriage, were thus often justified using information that lacked any real technical detail but at least appeared like cutting‐edge scientific research. In addition to making claims about sex and desire seem rational and objective, it also sold to readers the idea that a particular, restrictive kind of marital sexual relationship was the desirable outcome of natural and normal bodily processes.

Such diverse content is not only testament to the various scientific theories around sexual difference developing and circulating at this time. I would also argue that it reflects the different backgrounds of the authors themselves. While historians of sexology have emphasised the growing influence of specialist sexual scientists, often seeing advice as the endpoint of a process in which elite sexological knowledge was simplified and disseminated to the public, it was actually not especially common for texts in my sample to make much of contemporary research by key sexological figures of the era. Though Vern Bullough has claimed that physicians were increasingly overlooked as sexual advisors from the turn of the twentieth century, large numbers of these mass‐produced texts were authored by those with general medical training, ostensibly drawing from their experiences of helping their patients through sexual issues.^55^ The version of sexual science produced by many of these authors often relied more on the increasing social and cultural power of physicians, general medical knowledge, and their patients’ questions and anxieties than it did on popularising specialised medicine or cutting‐edge sexological work.^56^ Howard Chiang's notion of a sexology defined by ‘expert heterogeneity’ resonates here, with clear evidence of ‘interactions and distinctions’ between medicine and science on display.^57^

While many of the authors had some kind of medical training, be it specialist or more commonly general, it is also worth noting that some of the figures claiming ‘scientific authority’ over matters of sex had no formal medical or scientific experience at all. Leo Markun, for example, (who wrote a range of popular texts on evolutionary biology and psychology) graduated with a degree in English Literature from Harvard, while *Sexology* authors included members of the clergy, novelists, amateur historians, artists and journalists, writing alongside sexologists and medical doctors. William J. Fielding (author of the popular ‘Sanity in Sex’ series of Little Blue Books published by Haldeman‐Julius) had a clerical background, and was employed as secretary for the Tiffany Company from 1909 until his retirement in 1963. This did not stop a reader assuming formal expertise, addressing him as ‘Dear Dr Fielding’ in correspondence that praised him for ‘clearing up some of the mysteries of … life’.^58^ Clearly, then, in these cases, authors’ claims to authority were not necessarily about particular forms of training, or a mastery of specific scientific knowledge. Instead, their claims to be ‘scientific’ – claims that were clearly taken seriously by readers – seem to have rested on the fact that they were in a position to provide information: information they claimed was ‘truthful’ and ‘factual’, couched in the general language and rhetoric of science. Therefore, this kind of popular sexology seems not to have been defined by a solid idea of what the science of sex was or by a coherent set of theories or approaches. Instead, authors often simply invoked the general power and authority of science to justify their claims and to validate their ability to speak about sex to the public.

While sex advice authors spent considerable time discussing how and why husbands and wives were different, their readers were not only interested in hypothetical discussions of sex and marriage. It is clear from readers’ letters that specific instructions about sexual activities and techniques were much in demand, especially as the (tricky for many to achieve) mutual, simultaneous orgasm was increasingly touted in prescriptive literature as the most important outcome of sexual activity.^59^ One correspondent, for example, wrote to Fielding to express anxiety about his ‘decidedly curved’ penis and ‘put it in + let it lay’ technique, asking for further clarification from the author about the ideal ‘duration of the in + out motion’ to guarantee a mutual orgasm.^60^ The nature of the texts authors like Fielding were producing – designed to provide a ‘how‐to’ for harmonious marital living – therefore meant that apparently ‘scientific’ notions of sexual difference also had practical applications. While it is difficult to get a comprehensive sense of how seriously readers would have taken this advice or how it would have played out in different marital bedrooms, in the texts notions of sexual difference were evidently not just discussed as abstract concepts. Rather, diverse and often imprecise assertions about innate, biologically determined sexual difference were put forward as directions for actual, embodied sexual experience, and used to add authority to claims about what constituted acceptable sexual practice for both men and women.

Ideas of biologically and physiologically determined activity and passivity in men and women were used to argue that a man should play the most prominent and leading role in marital sex. While it was argued that women were powerfully sexual beings, as the natural sexual aggressor men were encouraged to take the lead in increasingly elaborate lovemaking and given the responsibility for rousing his wife's sexuality through skilful sexual technique. These responsibilities included activities like foreplay. As Peter Laipson and Steven Seidman have shown in their rich accounts of changing attitudes towards pre‐coital erotic activity in American advice, discussions of foreplay were not a feature of nineteenth‐century sex and marriage guides; however, their research has found that by the 1920s, it was seen as vitally important to a satisfying sexual life.^61^ Sex, Laipson argues, was no longer seen as a ‘single event but as a series of acts whose satisfying consummation required careful activity at every step’^62^; a conclusion supported by contemporary advice authors’ claims that ‘Foreplay is not to be set off as separate from the supreme love‐act of coitus’.^63^ As with the broader, more theoretical discussions of sexual difference outlined above, authors claimed scientific authority to prescribe appropriate pre‐coital activities in highly gendered terms, tying specific behaviours to assumptions about the particular, complementary sexual natures of husbands and wives. Men were said to have a particularly important role to play the early processes of lovemaking. As the natural, biologically defined sexual initiator, the male reader was told that he needed to take the initiative and work to woo his wife before every sexual encounter. Manuals therefore regularly included diagrams or descriptions of female genitals to guide them, describing the function of the clitoris as the source of female pleasure, and outlining the importance of ‘preliminary stimulation of the woman in love‐play’ to ensure she was aroused enough for comfortable penetration.^64^ While some advice was oblique, often guides were fairly explicit, especially in more expensive texts bought through mail order, or protected from the censors with claims they were for the eyes of physicians or other professional men only.^65^ Robinson's 1922 *Married Life and Happiness*, for example, outlined some of the different erogenous zones of a woman, and encouraged husbands to caress breasts, lips, hair and armpits ‘as a preliminary to the sexual act proper’.^66^ While women were not completely free of responsibility for pre‐coital activities (Walter Robie encouraged wives to take their husbands to ‘seventh heaven’ through ‘meaningful kisses’, for example), authors from a range of backgrounds argued that the active and passive roles of men and women were biologically mandated and translated into specific sexual functions.^67^ Men were expected to pursue sexual activity, to initiate contact and to lead an encounter; women were expected to first resist and then respond to the stimulus, before welcoming and reciprocating her husband's sexual advances.

It is clear from these examples that the power of science was frequently invoked to justify notions of sexual difference in these texts. It did not matter that the scientific content of many of these works was ill‐defined or imprecise or even produced by figures who had no formal scientific training at all. By grounding ideas about the innate sexual differences between men and women in assertions about ‘truthful’, ‘factual’ and ‘scientific’ ideas about the complex workings of the body, authors were able to make them seem rational, factual, but also inevitable; simply a part of the biological make up of a normal man or woman. Neuhaus has cautioned against oversimplifying the representations of gender and sexuality in contemporary sex manuals, arguing that they ‘acknowledged women's political gains while simultaneously reasserting male privilege’.^68^ Clear from my analysis here is how a diverse range of authors attempted to harness the language and power of science to facilitate such contestations around gender. While numerous authors took the time to criticise the negative impact of a male‐led society on women, and to denounce unequal or oppressive marital arrangements as regressive or outmoded, they nevertheless produced advice that said sexual passivity and a lack of sexual agency was an inescapable factor of female nature.

While authors were making practical suggestions, it is, of course, impossible to know how such theories were used, adapted or defied in actual American bedrooms. As historians such as Michiko Suzuki have shown, readers of scientific advice texts were not devoid of agency and undoubtedly adapted the information they were consuming to meet their own needs and agendas; ignoring some points, listening to others and negotiating everything else to fit in with their own specific inclinations, desires and anxieties. However, at a time when there were real, widespread concerns about issues like the increasing power and independence of women, falling birth rates and high numbers of women choosing not to marry, such an approach can be seen to have supported broader patriarchal notions of male superiority. Even in marriages sold as fairer, happier and more companionate, women were told that the innate workings of their bodies and minds determined that they should be subordinate in the bedroom. Advice writers drew quite liberally on different scientific approaches and theories to show, for instance, that the ‘source of erotic pleasure in the case of the male lies in activity, but in the female in the passive state’, emphasising that ‘sexual subordination is a necessary element in the sexual enjoyment of women’.^69^ As Robinson neatly summarised, even when living the ‘new’, more equal heterosexual ideal, it was better for women to accept their inevitable subordinate role than to fight it: ‘This is the way man and woman have been made by nature … the differences lie in biological roots, and it is futile to fight and rail against nature and biology’.^70^

As we have seen, the actual content of popular scientific advice was often vague and devoid of much technical content. Nevertheless, science – a term often bunched together with objective sounding descriptors like ‘rational’ and ‘sane’, and with promises that it would help in ‘destroying sexual error and spreading sexual truth’ – was clearly central to discussions in many contemporary advice texts about what appropriate sexuality should look like and how it should be performed.^71^ As this article has so far shown, authors claimed that to achieve a ‘good’, ‘normal’ marriage men and women should live out their respective, appropriate sexual roles through specific behaviours; actions that would generally follow a ‘natural’ order defined by the workings of a sexed body, leading to a mutually pleasurable, healthful and harmonious married life. ‘Natural, regular, mutual sex participation’, Robie argued, would do much to ensure ‘health and longevity in both the contracting parties’.^72^ Of course, the flip side of this approach was that, in addition to giving authors the authority to pontificate on what ‘good’ sex looked like, it also gave them room to argue that any actions that fell outside of their prescriptions should be cast as perverse or potentially pathological. The list of activities that were depicted as being detrimental to the health of the body and mind, or otherwise possibly symptoms of underlying sexual perversity, was an extensive one for both sexes: masturbation, the use of prostitutes, a lack of foreplay, engaging in extra‐marital sex, sadism and masochism and improper bodily hygiene were all touted as undesirable or potentially injurious, for example. Failure to live up to gendered expectations around sexual behaviour also fell into this category, as authors spent significant time warning their readers that an inability to perform their specific sexual roles could either be a worrying symptom of myriad kinds of diseases or else could lead to one. Readers were therefore presented with a version of sexual science that not only authoritatively dictated what constituted good sex, but could also be marshalled to argue that certain sexual behaviours should be considered abnormal, harmful or degrading.

Against a backdrop of broader attempts to regulate female sexuality occurring at this time, it is perhaps unsurprising that authors paid particular attention to the appropriate sexual behaviour of women.^73^ By framing these discussions as being about the science of the body, authors were able to assert that wives who did not adequately perform their expected sexual role were not only at risk of an unhappy marriage, but that there could be dire consequences for their health. As one contributor to *Sexology* magazine asserted in an article on sexual difficulties, ‘Nature, when we try to violate her laws, always takes some revenge’.^74^ Women, for example, who flouted expectations of passivity, were too sexually independent of their husbands, or demonstrated sexual desire outside of the confines of the marital bed were described as ‘sexually abnormal’ or pathologically oversexed.^75^ One writer argued while it was not wrong for women to experience strong sexual feelings, ‘nymphomania’ was a possible diagnosis for women who felt sexual desire too intensely.^76^ The author claimed ‘unusual glandular conditions’ were to blame for women who desired sex without an emotional connection with a man who was not her companion or protector, or who might pursue multiple sexual relationships outside of marriage. They argued that like homosexuals such women should be pitied rather than censured, given that their particular affliction was a hormonal disorder – a chemical imbalance that manifested itself in abnormal and adverse sexuality.^77^ Other authors were less sympathetic, arguing that women who suffered from ‘abnormal erotism’ tended to display criminal tendencies as well as inclinations towards violence or adultery.^78^ The kind of ‘hypersensual’ woman depicted here was said to be not only a danger to herself but also a ‘great danger to the health and even the very life of her husband’.^79^ Many advice writers claimed, then, that contravening expectations of sexual passivity, or defying a wife's ‘naturally’ subordinate sexual role, was not just a moral failing. Instead, by tying it to apparent inevitabilities of the body, they grounded their critique of powerful female sexuality using the power and authority of science, arguing that it was both the potential cause and effect of a disorder or disease.

But the majority of discussion around problematic female sexuality in popular advice was not focused on women who were too sexual, but rather on those who were not sexual enough. New, eroticised marital ideals about the importance of sex and mutual pleasure now challenged older, more conservative conceptualisations of women's sexual nature that saw them as inherently less sexual than men.^80^ While prescriptive texts made claims about the innate, natural passivity of women they nevertheless argued that women did have a potent sexuality that required regular expression and release: Robinson claimed, for example, that in women there existed a ‘strong sex instinct’ that needed ‘regular natural gratification’.^81^ Married women were therefore expected to walk a rather thin line: while they needed to be coy, passive and not too sexually independent, they were also expected to be a willing sexual partner, highly responsive to their husband's careful sexual attention and able to consistently reach orgasm. Women who were too passive or resistant in the marital bedroom were now cast as being sexually cold or anaesthetic – authors pathologised inadequate sexual desire in women by diagnosing them as suffering from ‘frigidity’, a disorder Peter Cryle and Alison Moore have shown was of increasing interest in medical and sexual scientific circles in the early twentieth century.^82^

Some advice discussed frigidity as a congenital absence of sexual desire in women, often linking a failure to respond to sexual stimulus to ‘structural’ causes such as hormonal or glandular defects, or other ‘abnormalities and chronic affections of the generative organs’.^83^ However, for the most part advice texts described frigidity as a learned disorder with two key causes. Authors frequently asserted that a woman's lack of desire or reluctance to respond to her husband's sexual advances was the result of years of conditioning, the outcome of living in a society that had trained women to see sex as somehow degrading. For instance, one text argued that frigidity was ‘cultivated by a process of miseducation with respect to the vital problems of life, to which a large number of refined women in particular have been subjected’.^84^ Describing frigidity as ‘largely a mental twist, a personal deviation from the normal’, Elwood L. Fantis argued that frigidity was not encountered in ‘savage tribes’ and was the result of modern society's repressive sexual mores; a conclusion mirrored by David H. Keller, who stated that a lack of frigidity in ‘savages’ was proof that it was ‘to a large extent…linked with modern civilisation’.^85^ Women suffering from this kind of frigidity were told that the answer to this issue was fairly simple, and that they just needed to accept modern sexual values, and embrace their natural sexuality. Fielding, for example, called on the frigid wife to ‘recognize her function and be an active participant’ in marital sex; to ‘remember her humanity and judiciously keep in control, but not persistently suppress, her sexual prompting’.^86^

While frigidity as a disorder was sometimes linked to a woman's failure to recognise or perhaps attempts to suppress what was described as her own naturally powerful sexuality, blame was also often levelled at sexually incompetent husbands. Frigidity manifesting as a lack of sexual desire, the inability to have penetrative sex, an aversion to frequent sexual expression or failing to consistently reach orgasm were all attributed to a man's inadequacy at playing the role of the sexual aggressor. An anonymous *Sexology* author asserted that in most cases of frigidity brought to doctors, ‘the trouble usually is in the husband … the husband just doesn't know how to woo his wife properly’.^87^ Authors emphasised that in not living up to expectations about his ability to arouse his partner, a husband was the root cause of most cases of frigidity, with a lack of sexual satisfaction and harmony leading to feelings of sexual antipathy in wives. The answer to this was quite simple in theory, though authors recognised it might be trickier in practice: a husband needed to fulfil his biological role as sexual leader and initiator by improving his sexual technique, spending more time on ‘preliminary wooing’, and constantly working to show ‘the consideration which the feminine nature unconsciously, but uncompromisingly, demands.^88^ Readers were told that this was high‐stakes stuff, as failing to deal with frigidity and thus living a sexless married life was seen to have significant and worrying ramifications. Wood called it a ‘physiological crime’, stating that people who lived an abstinent married life were ‘warped, twisted out of normal humanness … afflicted with ingrowing sex, as unnatural as an ingrowing nail’, living counter to the ‘deep physiological demands implanted in every individual’.^89^

Seemingly objective notions of biologically determined sexual difference, as well as rhetoric around sex and its apparently close relationship with health and wellbeing, were therefore frequently used to construct ideas about what constituted acceptable sexuality in early twentieth‐century sex advice. On the one hand, such an approach allowed authors to claim their prescriptions were modern, progressive and objectively positive. By framing their advice as ‘scientific’ in different ways, they appeared on the surface to throw off older taboos around sex, encouraging readers to embrace a more satisfying sexual life that promised to better serve the needs of both mind and body. On the other hand, however, these ideas may have been strategically deployed in reality, such ‘scientific’ prescriptions served to create quite tightly gendered definitions of desirable sexuality tied to assumptions about the specific, fixed workings of sexed bodies. Although generally lacking in technical scientific detail, scientific ideas and rhetoric nevertheless suffused this kind of advice, helping to construct and authorise a ‘new’, innovative heterosexual marital ideal that in many ways continued to uphold an older, more conservative sexual agenda.

The first half of the twentieth century saw some profound changes to marital advice. These changes came as a response to a moment of great upheaval and anxiety: the era saw increasing feminist agitation and changes to the social and political power of women, a falling birth rate amongst the white middle classes, and widespread concerns about the failures of orthodox marriage. In this context, readers were told that not only should marriage be saved but that it could be fairer, happier and more companionate than ever before. Sex was not something distasteful to be skirted around gently but rather something that needed to be handled head on, placed right at the heart of these promises. Advice authors claimed that sex done ‘right’ could make people happier and healthier. But, more than that, they claimed it could all but guarantee long and lasting marriages, the stability of the family, and indeed of American society as a whole. As this article has shown, authors of prescriptive literature worked with quite restrictive notions of sexual difference when preaching such visions of modern marriage. These notions served to uphold the idea that acceptable sexuality could only ever be marital and heterosexual, and, ultimately, that women should be subordinate in the bedroom. Carolyn Herbst‐Lewis has shown how notions of science and health produced ‘naturalized visions’ of gender and sexuality that were difficult to challenge in the Cold War context, tied to concerns about citizenship and identity.^90^ But my research here has shown that ‘science’, in different ways, played an important role in underpinning such assertions in the early part of the century too. In particular, I have demonstrated that by tying arguments about appropriate sexual behaviour to ideas about the workings of the sexed body, the power of science was invoked to make sexual difference – and thus ideas about the dominant and subordinate sexual roles of men and women – appear new, progressive, and wholly rational, but also natural and immutable.

As analysis has shown, though, the version of sexual science on display in these texts was a dynamic and often indistinct one. The science of sex in the mass‐produced advice discussed here was certainly not just a top down ‘popularization’ of contemporary sexological research; nor was it just information about venereal disease and sexual selection, or simply a cynical tool in the campaign for so‐called positive eugenics. Instead, we can see that it was made up of information and ideas from a broad range of different fields, discussing both the theory and practice of sex. Authors cherry‐picked data, approaches and rhetoric from old and new research and presented this alongside their own experiences in order to make their points. While some texts did look to contemporary sexological work, many – especially those by medical doctors and ‘amateur’ sexologists like Fielding – relied on quite broad, general medical knowledge and information gleaned from their own correspondents. Many, though, appeared to have used promises of science in even less defined or structured ways. In numerous cases, ‘science’ acted not as a marker of specific knowledge but as an apparent rejection of embarrassment and salaciousness – a claim to rationality and objectivity on a subject that was still taboo to many. Claiming ‘scientific’ authority over sexual topics, authors from diverse backgrounds appealed to readers and legitimised talking about sex in public by framing their work as ‘factual’ information about sex, delivered in ‘plain, honest, scientific language’.^91^

In many ways, then, this article is an early intervention into what needs to be a much bigger conversation about what it meant to talk ‘scientifically’ about sex in public at this time. Further explorations are needed around where scientific authority came from and who had it, as well as how ‘popular sexology’ responded to, built from or challenged other kinds of sexual expertise at this time. There is certainly more work to be done to better understand this ‘popular’ sexual science before the ‘watershed’ moment of mid‐century, and to interrogate the complex and diverse ways science shaped and was shaped by sexual culture in early twentieth‐century America.

## Notes

1 See Jonathan Ned Katz, *The Invention of Heterosexuality* (Chicago: University of Chicago Press, 2007); Peter Laipson, ‘“Kiss Without Shame, for She Desires It”: Sexual Foreplay in American Marital Advice Literature, 1900–1925’, *Journal of Social History* 29 (1996), pp. 507–25; Jennifer Terry, *An American Obsession: Science, Medicine, and Homosexuality in Modern Society* (Chicago: University of Chicago Press, 1999), p. 120.

2 See Jeffrey P. Moran, *Teaching Sex: The Shaping of Adolescence in the Twentieth Century* (Cambridge and London: Harvard University Press, 2000), p. 94. See also Lawrence R. Samuel, *Sexidemic: A Cultural History of Sex in America* (Lanham: Rowman & Littlefield, 2013), p. 10.

3 Clement Wood, *The Evolution of Sex* (Girard: Haldeman‐Julius, c. 1920s).

4 John Olszowka, Marnie M. Sullivan, Brian R. Sheridan and Dennis Hickey, *America in the Thirties* (New York: Syracuse University Press, 2014), esp. pp. 166–201; Sarah Jane Deutsch and Nancy F. Cott, *From Ballots to Breadlines: American Women, 1920–1940* (Oxford: Oxford University Press, 1998).

5 Terry, *An American Obsession*, pp. 125–6. On the relationship between race and contemporary discussions of marriage see Julian Carter, *The Heart of Whiteness: Normal Sexuality and Race in America, 1880–1940* (Durham: Duke University Press, 2007); Melissa N. Stein, *Measuring Manhood: Race and the Science of Masculinity, 1830–1934* (Minneapolis: University of Michigan Press, 2015).

6 Jessamyn Neuhaus, ‘The Importance of Being Orgasmic: Sexuality, Gender, and Marital Sex Manuals in the United States, 1920–1963’, *Journal of the History of Sexuality* 9 (2000), pp. 447–73, here p. 449.

7 On sex advice and positive eugenics, see Wendy Kline, *Building a Better Race: Gender, Sexuality, and Eugenics from the Turn of the Century to the Baby Boom* (Berkeley: University of California Press, 2001). On Freud and psychoanalysis, Linda Gordon, *The Moral Property of Women: A History of Birth Control Politics in America* (University of Illinois Press, 2002), p. 125; Lawrence R. Samuel, *Shrink: A Cultural History of Psychoanalysis in America* (University of Nebraska Press, 2013). On the rising influence of sexology, see Terry, *An American Obsession*, p. 120.

8 See, for example, Neuhaus, ‘The Importance of Being Orgasmic’, p. 451; Christina Simmons, *Making Marriage Modern: Women's Sexuality from the Progressive Era to World War II* (Oxford: Oxford University Press, 2009), pp. 187–9.

9 Miriam G. Reumann, *American Sexual Character: Sex, Gender, and National Identity in the Kinsey Reports* (Berkeley: University of California Press, 2005), p. 6.

10 Janice Irvine, *Disorders of Desire: Sexuality and Gender in Modern American Sexology* (Philadelphia: Temple University Press, 2005); Reumann, *American Sexual Character*. See also Carolyn Herbst Lewis, *Prescription for Heterosexuality: Sexual Citizenship in the Cold War Era* (University of North Carolina Press, 2010). On early American sexual science, see Greta LaFleur, *The Natural History of Sexuality in Early America* (Baltimore: Johns Hopkins University Press, 2020); Benjamin Kahan, ‘The Unexpected American Origins of Sexology and Sexual Science: Elizabeth Osgood Goodrich Willard, Orson Squire Fowler, and the Scientification of Sex’, *History of the Human Sciences* 34 (2020) https://doi.org/10.1177/0952695120910051

11 See, for example, Stein, *Measuring Manhood*, esp. chapter 3; Laura Tarzia, ‘From Marriage Manuals to Mars and Venus: Darwin, Sex Advice, and the Promotion of Inequality’, *Women's Studies* 44 (2015), pp. 368–91, here p. 375.

12 Laura Doan, ‘Troubling Popularization: On the Gendered Circuits of a “Scientific” Knowledge of Sex’, *Gender & History* 31 (2019), pp. 304–18, here p. 305.

13 Mary Fissell and Roger Cooter, ‘Exploring Natural Knowledge: Science and the Popular’, in R. Porter (ed.), *The Cambridge History of Science* (Cambridge: Cambridge University Press, 2003), p. 156.

14 For an account of the sexual materials available through mail order at this time, see Jay Gertzman, ‘“Esoterica” and “The Good of the Race”: Mail‐Order Distribution of Erotica in the 1930s’, *The Papers of the Bibliographical Society of America* 86 (1992), pp. 295–340.

15 Details on sales figures are outlined in E. Haldeman‐Julius, *The First Hundred Million* (New York: Simon and Schuster, 1928), p. 17.

16 Jay A. Gertzman, *Bookleggers and Smuthounds: The Trade in Erotica, 1920–1940* (Philadelphia: The University of Pennsylvania Press, 2002), p. 55. Little historical work has been done on the Little Blue Books series, with the exception being Eric Schocket, ‘Proletarian Paperbacks: The Little Blue Books and Working‐Class Culture’, *College Literature* 29 (2002), pp. 67–78. On the difficulties of referencing Haldeman‐Julius's publications, see Richard Colles Johnson and G. Thomas Tanselle, ‘Haldeman‐Julius “Little Blue Books” as a Bibliographical Problem’, *The Papers of the Bibliographical Society of America* 64 (1970), pp. 29–78.

17 Maxwell Vidaver, ‘The Reason for Sexology’, *Sexology* (1933), p. 3.

18 James Oppenheim, *The Common Sense of Sex* (Girard: Haldeman‐Julius, 1926), p. 3.

19 Laipson, ‘Kiss Without Shame, For She Desires It’, pp. 512–13.

20 Max Saunders, *Imagined Futures: Writing, Science, and Modernity in the To‐Day and To‐Morrow Book Series, 1923–31* (Oxford: Oxford University Press, 2019), p. 170.

21 Kline, *Building a Better Race*, p. 156.

22 Max J. Exner, *The Sexual Side of Marriage* (London: George Allen & Unwin Ltd, 1923), p. 25.

23 Thomas Laqueur, *Making Sex: Body and Gender from the Greeks to Freud* (Cambridge: Harvard University Press, 1992); Cynthia Eagle Russett, *Sexual Science*: *The Victorian Construction of Womanhood* (Cambridge: Harvard University Press, 1989); Kimberly A. Hamlin, *From Eve to Evolution: Darwin, Science, and Women's Rights in Gilded Age America* (Chicago: University of Chicago Press, 2014).

24 Laipson, ‘Kiss Without Shame, For She Desires It’.

25 Wilfred Lay, *Confidential Chats with Husbands* (Girard: Haldeman‐Julius Company, 1925), p. 13.

26 William Fielding, *Women's Sexual Life* (Girard: Haldeman‐Julius Company, 1925), p. 5.

27
*Sexology*, February 1934, back cover.

28 William Fielding, *Woman: The Eternal Primitive* (Girard: Haldeman‐Julius Company, 1927), p. 15.

29 Peter Stearns, *Battleground of Desire: The Struggle for Self‐Control in Modern America* (New York; London: New York University Press, 1999), p. 197.

30 Fielding, *Woman's Sexual Life*, p. 3.

31 Wood, *Evolution of Sex*, p. 28.

32 William Fielding, *What Every Married Man Should Know* (Girard: Haldeman‐Julius Company, 1924), p. 21.

33 Exner, *The Sexual Side of Marriage*, p. 23; Robinson, *Married Life and Happiness, or Love and Comfort in Marriage* (New York: Eugenics Publishing Company: 1922), p. 36.

34 Joseph Greer, *The Physiology of Sex Life* (Girard: Haldeman‐Julius Company, 1925), p. 23.

35 Fielding, *What Every Married Man Should Know*, p. 20.

36 See, for example, Oppenheim, *The Common Sense of Sex*, p. 19.

37 Fielding, *Woman's Sexual Life*, p.3.

38 Oppenheim, *Common Sense of Sex*, p. 19.

39 Greer, *The Physiology of Sex Life*, p. 8.

40 Oppenheim, *Common Sense of Sex*, p. 16.

41 Exner, *The Sexual Side of Marriage*, pp. 84–5.

42 Wood, *The Evolution of Sex*, p. 26.

43 Wood, *The Evolution of Sex*. On the links between human and animal sexuality in evolutionary biology, see Kimberly A. Hamlin, ‘The Birds and the Bees: Darwin's Evolutionary Approach to Sexuality’, in Jeannette Eileen Jones and Patrick B. Sharp (eds), *Darwin in Atlantic Cultures: Evolutionary Visions of Race, Gender and Sexuality* (London: Routledge, 2010), pp. 53–72.

44 Wood, *The Evolution of Sex*.

45 Hamlin, *Eve to Evolution*, p. 3. See also Susan Sleeth Mosedale, ‘Science Corrupted: Victorian Biologists Consider “The Woman Question”’, *Journal of the History of Biology* 11 (1978), pp. 1–55.

46 Nathan Q. Ha, ‘The Riddle of Sex: Biological Theories of Sexual Difference in the Early Twentieth‐Century’, *Journal of the History of Biology* 44 (2011), pp. 505–46.

47 On metabolic theories of sexual difference and the influence of Geddes and Thompson in the nineteenth century, see Cynthia Eagle Russett, *Sexual Science*, pp. 89–92; Gail Bederman, *Manliness and Civilization: A Cultural History of Gender and Race in the United States, 1880–1917* (Chicago: University of Chicago Press, 1996), p. 153; Jill Conway, ‘Stereotypes of Femininity in a Theory of Sexual Evolution’, *Victorian Studies* 14 (1970), pp. 51–2.

48 William Fielding, *Man's Sexual Life* (Girard: Haldeman‐Julius Company, 1925), p. 28.

49 Fielding, *Man's Sexual Life*, p. 28.

50 On the growing importance of hormonal research in this period, see Anne Fausto Sterling, *Sexing the Body: Gender Politics and the Construction of Sexuality* (New York: Basic Books, 2000), p. 150–51; Christer Nordlund, ‘Endocrinology and Expectations in 1930s America: Louis Berman's Ideas on New Creations in Human Beings’, *British Journal for the History of Science* 40 (2007), pp. 83–104; Michael Pettit, ‘Becoming Glandular: Endocrinology, Mass Culture, and Experimental Lives in the Interwar Age’, *American Historical Review* 118 (2013), pp. 1052–76.

51 William Lemkin, ‘Glands – The Determinants of Sex’, *Sexology*, Summer 1933, p. 21.

52 Lemkin, ‘Glands’, pp. 22–3.

53 Robinson*, Sex Knowledge for Men* (New York: Critic and Guide Co., 1922), p. 22.

54 Exner, *Sexual Side of Marriage*, p. 85.

55 Vern Bullough, ‘American Physicians and Sex Research and Expertise, 1900–1990’, *Journal of the History of Medicine and Allied Sciences* 52 (1997), p. 244.

56 Bullough, ‘American Physicians and Sex Research and Expertise, 1900–1990’, p. 244. On the growing power of American physicians, see Leighton E. Chuff, ‘America's Romance with Medicine and Medical Science’, *Daedalus* 115 (1986), pp. 137–59.

57 Howard Chiang, ‘Historicizing the Emergence of Sexual Freedom: The Medical Knowledge of Psychiatry and the Scientific Power of Sexology, 1880–1920’, *Gender Forum* 24 (2009), p. 4.

58 Letter from W.E. to William Fielding, 22 September 1926, William John Fielding Papers TAM.069, Box 1, Folder 1B, Tamiment Library and Robert F. Wagner Labor Archive, Elmer Holmes Bobst Library, New York University, New York.

59 On increasing importance of orgasm, see M. E. Melody and Linda M. Peterson, *Teaching America about Sex: Marriage Guides and Sex Manuals from the Late Victorians to Dr. Ruth* (New York: New York University Press, 1999), p. 113.

60 Letter from A.M.C. to William Fielding, 26 May 1921, William John Fielding Papers TAM.069, Box 1, Folder 7, Tamiment Library and Robert F. Wagner Labor Archive, Elmer Holmes Bobst Library, New York University, New York.

61 Laipson, ‘Kiss Without Shame, For She Desires It’, p. 508; Steven Seidman, *Romantic Longings: Love in America, 1830–1980* (New York: Routledge, 1991), p. 97.

62 Laipson, ‘Kiss Without Shame, For She Desires It’, p. 509.

63 Exner, *The Sexual Side of Marriage*, p. 103.

64 Exner, *The Sexual Side of Marriage*, pp. 60–64.

65 Despite the continued fervour of government censors well into the 1930s, Peter Laipson argues that even those texts that protected themselves from the censors with claims they were ostensibly only for the eyes of legal and medical professionals were in fact written for and available to a lay audience. Laipson, ‘Kiss Without Shame, For She Desires It’, p. 520.

66 Robinson, *Married Life and Happiness*, p. 62.

67 Robie, *Sex and Life: What the Experienced Should Teach and the Inexperienced Should Learn* (Boston: Gorham Press, 1920), p. 364.

68 Neuhaus, ‘The Importance of Being Orgasmic’, p. 454.

69 Fielding, *Woman's Sexual Life*, p. 13.

70 William J. Robinson, *Woman: Her Sex and Love Life* (New York: Eugenics Publishing Co., 1932), p. 326.

71 William J. Robinson, *Sexual Problems of To‐Day* (New York: Truth Publishing Company, 1921), preface.

72 Robie, *Sex and Life*, p. 402.

73 On the changing sexual values of early twentieth‐century America, see Kline, *Building a Better Race*, pp. 43–4; Mary E. Odem, *Delinquent Daughters: Protecting and Policing Adolescent Female Sexuality in the United States, 1885–1920* (Chapel Hill: University of North Carolina Press, 1995), p. 1.

74 ‘Penalties of Incomplete Sex Life, Part II’, *Sexology*, January 1936, p. 315.

75 Henrietta Lifsitz, ‘Sex – As It Seems to the Woman’, *Sexology* (1936), p. 214; Wood, *Physiology of Sex*, p 36.

76 W.S.P. M.D., ‘Nymphomania’, *Sexology* (1936), p. 445.

77 W.S.P., ‘Nymphomania’, p. 445.

78 Fielding, *Woman's Sexual Life*, p. 4.

79 Robinson, *Married Life*, p. 90.

80 For an overview of nineteenth‐century understandings of female sexuality, see Jacinthe Flore, *A Genealogy of Appetite in the Sexual Sciences* (Cham: Springer Nature, 2020), p. 60. See also Nancy F. Cott, ‘Passionlessness: An Interpretation of Victorian Sexual Ideology’, *Signs* 4 (1978), pp. 219–36; Carroll Smith‐Rosenburg, *Disorderly Conduct: Visions of Gender in Victorian America* (New York: Oxford University Press, 1985), pp. 197–216.

81 Robinson, *Married Life*, p. 36.

82 Peter Cryle and Alison Moore, *Frigidity: An Intellectual History* (Basingstoke: Palgrave Macmillan, 2011), pp. 6–7.

83 William Fielding, *What Every Married Woman Should Know* (Girard: Haldeman‐Julius Company, 1924), p. 60; Fielding, *What Every Married Man Should Know*, p. 37. On congenital frigidity, see also ‘Unsatisfied Wives’, *Sexology* (1938), pp. 833–4.

84 Fielding, *What Every Married Man Should Know*, p. 36.

85 Elwood L. Fantis, ‘Is Frigidity Inherited?’, *Sexology* (1934), p. 246; David H. Keller, ‘Frigidity is Often Curable’, *Sexology* (1935), p. 343. For further discussion of contemporary ideas about primitive sexuality, see Stein, *Measuring Manhood*; Andrew Lyons and Harriet Lyons, *Irregular Connections: A History of Anthropology and Sexuality* (Lincoln: University of Nebraska Press, 2004).

86 Fielding, *What Every Married Woman Should Know*, pp. 31–2.

87 ‘Unsatisfied Wives’, *Sexology* (1938), p. 833.

88 Fielding, *What Every Married Man Should Know*, p. 37.

89 Clement Wood, *Manhood: The Facts of Life Presented to Men* (Girard: Haldeman‐Julius Company, 1925), p. 48.

90 Herbst Lewis, *Prescription for Heterosexuality*, p. 5.

91 Robinson, *Sexual Problems of To‐Day*, p. 13.

